# Augmented reality for endoscopic transsphenoidal surgery: evaluating design factors with neurosurgeons

**DOI:** 10.1007/s11548-024-03225-9

**Published:** 2024-07-26

**Authors:** Jennifer Higa, Sonia Nkatha, Roxana Ramirez Herrera, Hani Marcus, Soojeong Yoo, Ann Blandford, Jeremy Opie

**Affiliations:** 1 UCLIC, University College London, London, WC1E 6BT UK; 2 WEISS, University College London, London, W1W 7TY UK

**Keywords:** Augmented reality, Neurosurgery, Minimally invasive surgery, Endoscopic transsphenoidal approach, Human–computer interaction

## Abstract

**Purpose:**

This study investigates the potential utility of augmented reality (AR) in the endoscopic transsphenoidal approach (TSA). While previous research has addressed technical challenges in AR for TSA, this paper explores how design factors can improve AR for neurosurgeons from a human-centred design perspective.

**Methods:**

Preliminary qualitative research involved observations of TSA procedures ($n=2$) and semi-structured interviews with neurosurgeons ($n=4$). These informed the design of an AR mockup, which was evaluated with neurosurgeons ($n=3$). An interactive low-fidelity prototype—the “AR-assisted Navigation for the TransSphenoidal Approach (ANTSA)”—was then developed in Unity 3D. A user study ($n=4$) evaluated the low-fidelity prototype of ANTSA through contextual interviews, providing feedback on design factors.

**Results:**

AR visualisations may be beneficial in streamlining the sellar phase and reducing intraoperative errors such as excessive or inadequate exposure. Key design recommendations include a lean mesh rendering, an intuitive colour palette, and optional structure highlighting.

**Conclusion:**

This research presents user-centred design guidelines to improve sensemaking and surgical workflow in the sellar phase of TSA, with the goal of improving clinical outcomes. The specific improvements that AR could bring to the workflow are discussed along with surgeons’ reservations and its possible application towards training less experienced physicians.

**Supplementary Information:**

The online version contains supplementary material available at 10.1007/s11548-024-03225-9.

## Background and introduction

The endoscopic transsphenoidal approach (TSA) is a minimally invasive neuro-surgical procedure conducted to remove tumours developing within and around the pituitary gland [1, 2]. The surgical approach to the pituitary is guided by the surrounding anatomy with aid from image-guided surgery (IGS) systems, such as neuronavigation, to visualise and localise critical landmark structures that are obscured from the endoscopic view by the anatomy and/or deformed due to the presence of a tumour [2]. The ‘Sellar Phase’ is particularly delicate as it involves navigating through an anatomically dense area with critical concealed anatomical structures that are in close proximity to the underlying tumour [3]; iatrogenic injury of structures such as the carotid arteries or optic nerves represent the worst-case scenario in TSA [1, 2]. Tasks in this phase require supplemental information from neuronavigation and an ultrasound doppler to localise obscured structures [1, 2].

A drawback of typical neuronavigation is the use of triplanar displays whose image slices require surgeons to construct, in their own minds, potentially complex 3D representations of anatomical and pathological structures [4, 5]. Moreover, triplanar displays require that surgeons stop operating momentarily, apply a probe to the region of interest (potentially near critical, delicate structures) and take their eyes off the surgical field to view the image guidance monitors, disrupting workflow [4]. While conventional IGS navigation systems have 3D renderings in addition to triplanar data, disruption to the workflow similarly manifests by virtue of probe use and a separate screen displaying the rendered images. Applying an IGS system that fuses virtual 3D anatomical models with the actual operating field, using augmented reality (AR), could enhance information support concerning obscured and/or deformed structures—aiding in orientation and facilitating precise, targeted surgery [3].

Most research focuses on the engineering challenges of registering virtual overlays with the operative scene or the efficacy of AR technology [6–8]. Although crucial, limited research explores the impact of human factors on the pragmatic and hedonic qualities of AR displays for intraoperative use [9, 10]. In this paper, we conducted two studies to explore how AR design conditions affect the acceptance, preferences, and usability of an AR-Assisted Navigation system for the TransSphenoidal Approach (ANTSA).

*Participants and Ethics*. Neurosurgeons trained in TSA with no prior experience using AR in surgical practice were recruited through convenience sampling. For Study 1, neurosurgeons in training were recruited (P1-P4) as with greater experience, surgeons develop practices that compensate for a lack of additional support mechanisms, reducing their need to rely on image guidance [4, 11]. Study 2 involved three neurosurgeons in training (P3, P5-P6) and one expert neurosurgeon (P7). Given the fundamental nature of qualitative research, our participant selection was intentionally small. This approach allowed for a focus on gathering rich, detailed, and complex insights into processes and workflows.

## Study 1: requirements gathering

Two observations of TSA and semi-structured interviews were conducted to explore neurosurgeons’ intraoperative information needs. Data was analysed thematically [12], and an AR mock-up of ANTSA was created and evaluated. It was evident that surgeons repeatedly sought to refine their understanding of the location and course of critical underlying structures, moreso in the steps preceding sella and dura exposure. Participants discussed using bony contours as a primary strategy for discerning the dense network of underlying structures—critically, the carotid arteries, the optic nerves and the optic chiasm. The carotids were mentioned in conjunction with the cavernous sinus—a venous structure which carries other blood vessels and nerves, and through which a segment of the carotids course. Participants emphasised the challenge in determining lateral and superior limits when bony contours are irregular or when sella bone is abnormally thick. They acknowledged the utility AR could provide in highlighting critical structures thereby aiding in determining exposure limits.

**Fig. 1:**
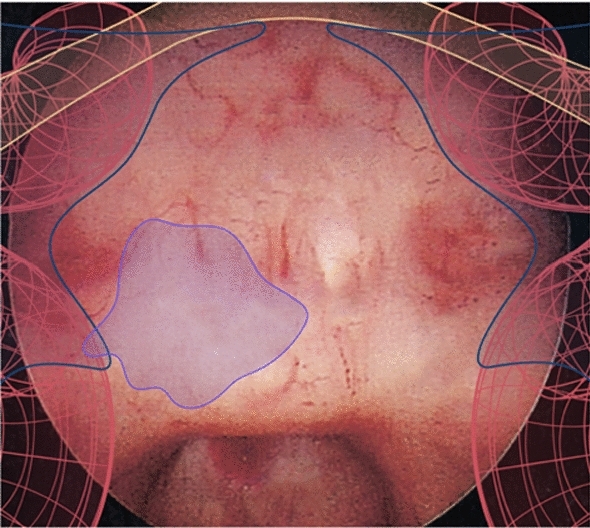
A mock-up of ANTSA illustrating critical anatomical structure and tumour overlay on the endoscope screen: carotids (red), cavernous sinus (blue), optic nerves (yellow), tumour (purple)

There were opposing viewpoints on sella exposure with three of the four surgeons favouring an optimal opening, determined by the tumour’s size and location. However, one surgeon was open to extending the sella as much as possible, prioritising instrument manoeuvrability. From these two competing perspectives, exposure during sellotomy should: (1) be wide enough to provide access to the tumour with surgical instruments, and (2), circumferentially limited to protect the surrounding structures.

Based on the synthesised information needs, an AR overlay mockup was developed in Reality Composer, an AR design tool, consisting of a static image of a patient’s sphenoid sinus and virtual representations of the carotid arteries, cavernous sinus, optic nerves, and tumour, as shown in Fig. 1. Participants were asked to give verbal feedback on how they would apply the AR tools intraoperatively. Participants remarked that AR would help guide their sellotomy as areas that are dangerous to expose would be more clear and area when the tumour is highlighted would more clearly demarcate where exposure is required. Notably, the surgeon who was keen to expose the sella more extensively, whilst using ANTSA reformed their strategy to a more optimal exposure. When faced with a scenario where the tumour was skewed to the left, their strategy involved focusing their exposure to the left to reduce unnecessary, risky exposure to the critical structures on the right. Thus, the user evaluation of a mock-AR overlay proved helpful in assisting surgeons to quickly interpret and counterbalance these competing interests. The results of this study are consistent with existing literature [3].

**Fig. 2:**
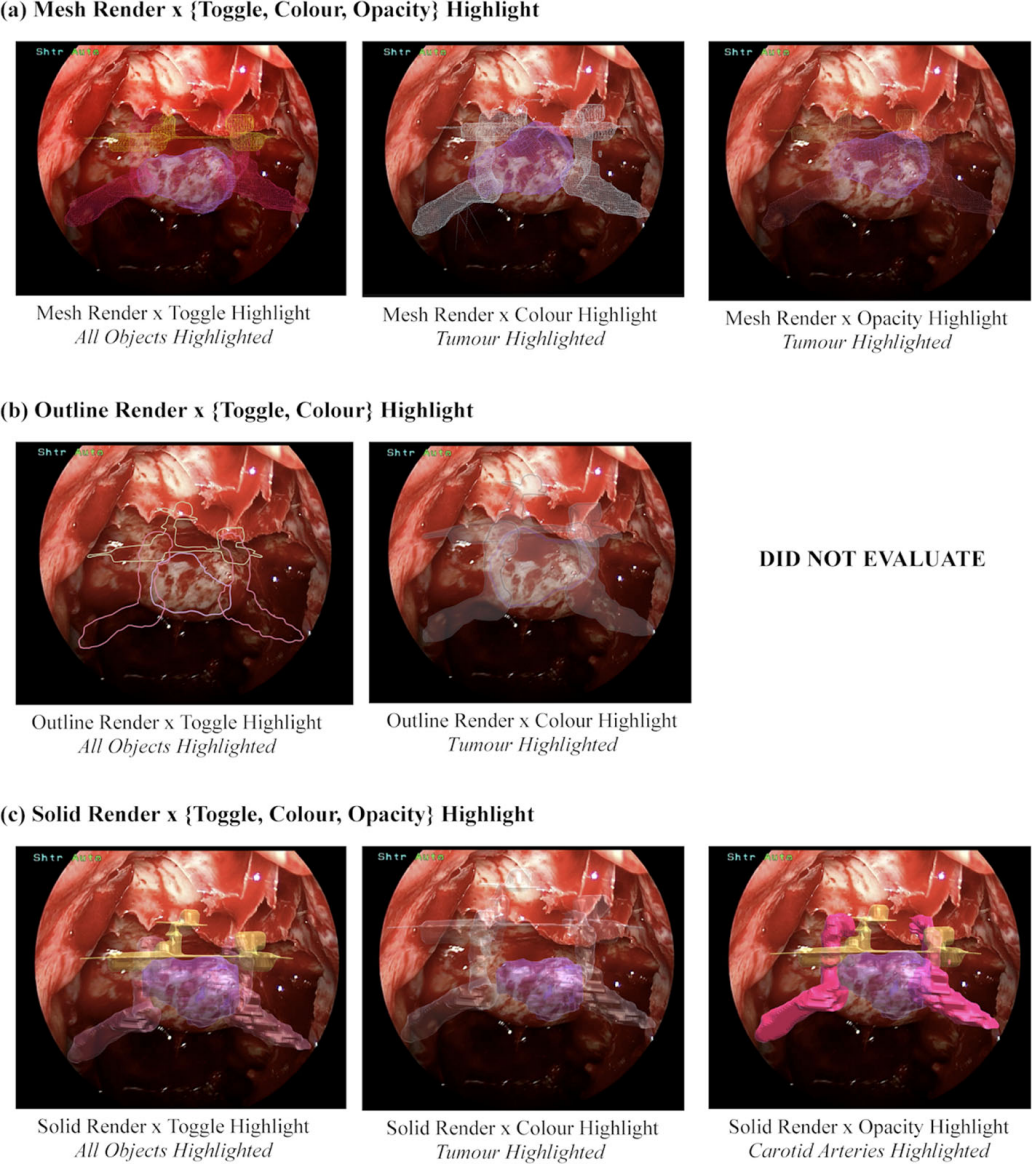
Examples of ‘*Render*’ and ‘*Highlight*’ design concepts used during Study 2

## Study 2: user evaluation

To expand our understanding of the design and interaction factors that may impact the usability of ANTSA we conducted a second study by developing and evaluating a low-fidelity prototype with design variations. Eight design combinations were collated into a $3 \times 3$ [Render Style x Highlight Style] matrix, of which examples of these can be seen in Fig. 2. The ‘*Render*’ style dictates how the 3D AR overlays are textured, while the ‘*Highlight*’ style dictates the way the 3D objects are displayed. 3D models of the anatomical structures of interest (tumour, carotid arteries, and optic nerves) were constructed using a patient’s preoperative MRI scan segmentation. These models were imported into Unity-3D, and manually aligned on a static image of a patient’s TSA endoscopic camera feed. A basic graphic user interface was developed for the primary navigational task, which involved the use of a digital pen to mark the area of sellar resection on-screen. While participants engaged in the primary task, they interacted with the 3D anatomical overlays using a footpedal, a common component in the medical domain, facilitating hands-free control of various devices. [13–15].

**Fig. 3:**
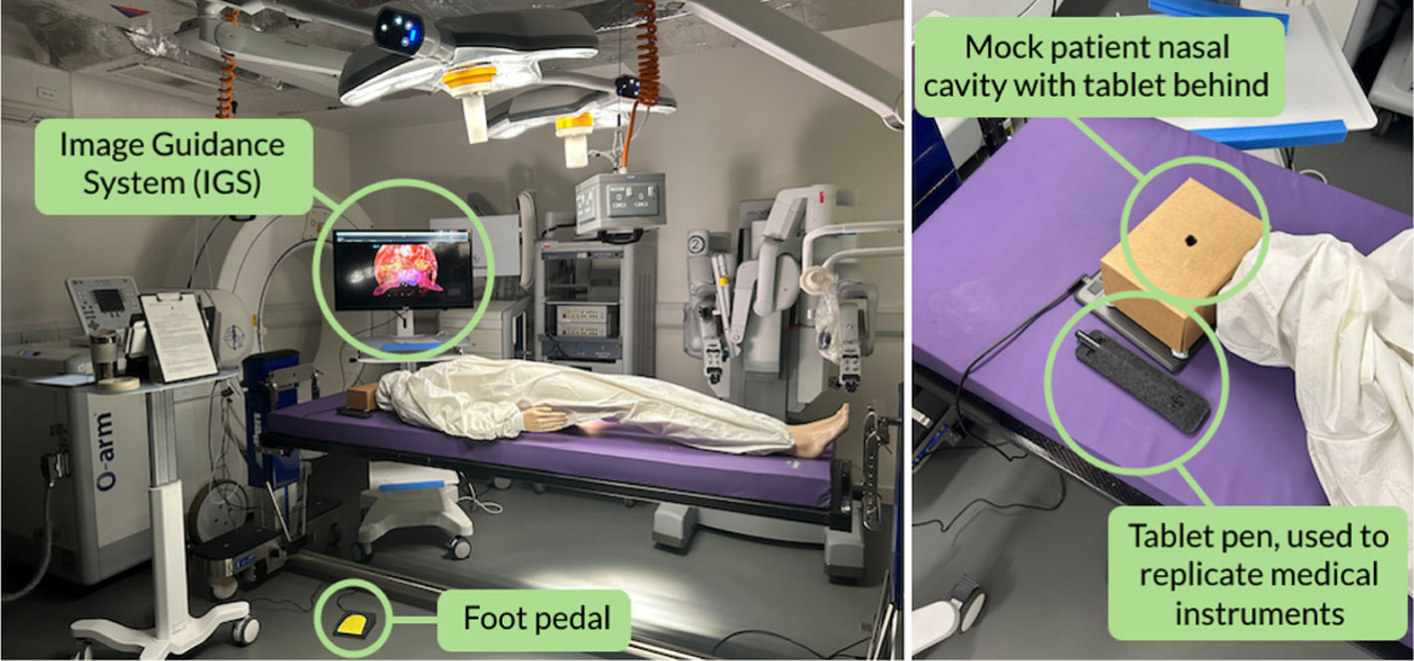
Mock OR setup for Study 2, showing the ANTSA concept prototype set up from the perspective of where the participant would stand (left), and from above, the tablet and pen used by participants to outline the target area, which is then displayed as a white outline on the monitor, indicating where the sellotomy would be performed (right)

Each press of the foot pedal highlights a different anatomical landmark (1: tumour; 2: carotids; 3: optic nerves; and 4: toggles all off). This allows the user to control the display of each 3D overlay at different stages of the procedure and visualise the spatial relationship between the three structures. The prototype was set up in a mock operating room (OR) in our lab to simulate the OR environment, based on observations of live TSA procedures at the hospital Fig. 3.

**Table 1:**
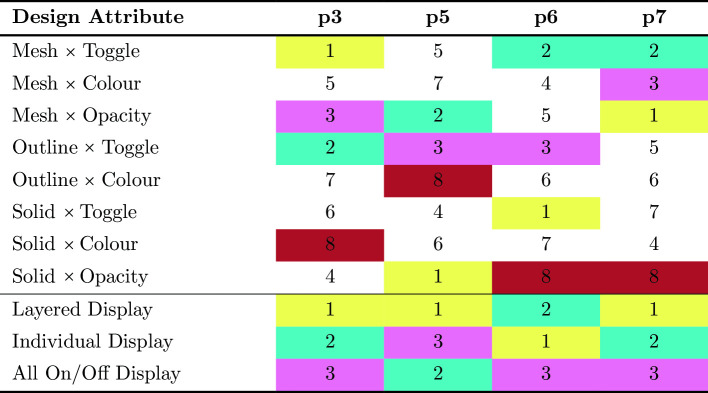
Design combos and foot pedal display rankings

Participants were given five minutes to familiarize themselves with the prototype. They then performed the primary task for each design combination while freely interacting with the 3D overlays. Combinations were provided in the same order as displayed in Fig. 2, mesh (a), outline (b), then solid (c) renders across all participants. Throughout this process, participants were asked to verbally express their thoughts in a think-aloud format. After the evaluations, they were asked to rank the visualisations on a digital ranking board followed by a semi-structured interview to gather additional feedback, analysed using thematic analysis [12].

### Results

The rankings showed little consensus on the “best” design combinations in regards to render style and highlight style; every participant ranked distinct design combinations as their “most preferable” combination (See Table 1). However, the pedal interaction display style did saturate on the “layered” approach as most preferable. The qualitative think aloud data revealed that the layered display allowed for surgeons to view the spatial relationship between the critical structures, which was important. The data also revealed several other insights; for example, our participants specified that only critical structures should be displayed; the AR should not obscure patient anatomy with solid renders; renders should be clear, detailed, and have distinct boundaries; and the colour scheme should match pre-existing schemes used in medical literature [16].

## Discussion and conclusion

Previous research alludes to AR streamlining surgical workflow in TSA [4, 5, 11]. The feedback from both studies has resulted in design guidelines that could streamline sella exposure, improving accuracy in tumour resection and critical structure avoidance, and reduce time spent looking away from the surgical field of view. Furthermore, participants expressed enjoying the implementation of AR to support neuronavigational guidance. Our studies have provided insight into the benefits of AR and how surgeons view future integration of AR intraoperatively for TSA.

Study 1 uncovers specific improvements AR could bring to the workflow in the ‘Sellar Phase’: (1) By adopting AR to visualise critical structures, surgeons would be less likely to cause unintentional damage to structures as they gain a more direct perception of the required boundaries; (2) with AR overlay of the tumour, surgeons are guided in optimally exposing the sella, ensuring that bony sella tissue is not resected excessively or inadequately; and (3) surgeons are keen to visualise the arteries at the beginning of the phase, when bony tissue is still intact, thereby facilitating the identification of arterial limits earlier in the phase and ensuring surgeons can implement strategies to protect the arteries earlier in the workflow. This would usefully complement the ultrasound doppler in identifying arterial margins, whose technology can fall short in detecting an arterial pulse with bony tissue concealing the carotids.

From Study 2, two main advantages were mentioned: Firstly, participants agreed that the surgeon knows the patient’s anatomy the best and that being able to see the true anatomy through the endoscope takes priority. Therefore, AR overlays would mostly be used as an adjunct to confirm their initial boundary scoping. Second, participants suggested AR visualisations be used for the training and education of neurosurgeons with less experience in TSA. Additionally, it was suggested that experts could use AR during operations to help explain their thought processes. However, the need for AR may decline for the individual as their experience in TSA increases.

The use of qualitative methods for conducting user studies in healthcare technology development involving highly specialised experts requires prioritizing in-depth understanding over quantitative breadth. User evaluations help to identify problems early on in the design process with most usability defects being discovered with sample sizes in the range of five to eight participants in formative evaluations [17, 18].

Due to the recruitment method used, the neurosurgeons in these studies were from the same hospital, which could have introduced bias. Moreover, due to the limited availability of senior clinicians and resource constraints, only one senior surgeon could participate in this study, potentially limiting the generalisability of our insights. However, the diversity in the overall training and practice backgrounds of all participants included adds a layer of representativity. Lastly, the order in which the design combinations were shown may have introduced bias and should be randomized in future studies. Nonetheless, future work will aim for broader generalisability by including participants from various hospitals. In future studies, we intend to address the issue of inattentional blindness [4, 5] and develop a high-fidelity ANTSA prototype situated within a real surgical setting to quantitatively assess improvements to surgical workflow, ensure proper integration with existing tools, assess the learning curve, and incorporate the complexities of live surgery.

## Supplementary Information

Below is the link to the electronic supplementary material.

Supplementary file 1 (pdf 381 KB)

Supplementary file 2 (mp4 80688 KB)

Supplementary file 3 (pdf 9696 KB)

Supplementary file 4 (png 51 KB)
