# Supplementary material for: Augmented reality for endoscopic transsphenoidal surgery: evaluating design factors with neurosurgeons
Source: Int J Comput Assist Radiol Surg. 2024 Jul 26;20(1):131–6. doi: 10.1007/s11548-024-03225-9 (PMC11759473; doi:10.1007/s11548-024-03225-9)

Appendix A – Study 2 Supplementary Materials

A.1 Interview Guide

Overview

Thank you for your participation. Today we will be evaluating several design factors and how it affects the usefulness of the AR overlay. This will be a contextual interview where we will ask you questions and to vocalise your thoughts as you interact with the prototype. This will be in the sellar phase so we are assuming that the endoscope is fixed.

This study consists of two parts: first part will be an evaluation of contour style and highlight style (which we will explain more about in a bit) and the second part will be an evaluation of only the pedal interactions in various forms, which we will also explain more about before beginning the second section.

Your task for each condition is to use the tool to indicate where you would conduct the sellar wall removal. Objective of the task is to expose the tumour as much as you can while avoiding critical anatomical structures. For the purposes of this study, we are focusing on the carotid arteries, optic nerves. Although the motion to outline the area does not mimic the motion you would use in reality, this is meant to replicate doing a task where you would rely on the endoscopic projection to help navigate. Please feel free to test out the this motion to familiarise yourself with it.

Highlight style will be controlled by the foot pedal you have here in front of you. Feel free to interact with it and familiarise yourself.

The location of each structure will differ slightly between conditions. This is so you are able to use the AR overlays to help guide you equally for each scenario. For all scenarios, please assume that the overlays are accurately located.

After part I and after pat II, you will be asked to do a ranking task on your preferences as well as a few questions.

Do you have any questions so far?

Part I: Task Conditions (8 Combinations of Contour Style and Highlight Style)

After each condition we will ask you to remove the tool from the pad so I can set up for the next task.

First we will begin with the:

Mesh overlay x [toggle, colour, opacity]

- Was there anything distracting?
- What were you thinking about when you were drawing the overlay?

- How easy is it to define the contours of the important structures using the AR overlay?
- Assuming the accuracy of the AR overlays, please indicate your confidence in the task with this design combination.
- Any other thoughts, feeling or feedback?

Outline overlay x [toggle, colour]

- How distracting are the AR overlays
- How easy is it to define the contours of the important structures using the AR overlay?
- Assuming the accuracy of the AR overlays, please indicate your confidence in the task with this design combination.
- Any other thoughts, feeling or feedback?

Solid overlay x [toggle, colour, opacity]

- How distracting are the AR overlays
- How easy is it to define the contours of the important structures using the AR overlay?
- Assuming the accuracy of the AR overlays, please indicate your confidence in the task with this design combination.
- Any other thoughts, feeling or feedback?

https://miro.com/app/board/uXjVM06Lres=/?share_link_id=91798650876

Post-Part I Interview Questions

- Rank which combination of contour style and highlight style according to preference from Best to Least.
  - Why did you choose x as rank #n?
- If you could improve your highest ranked overlay style, how would you improve it for your purposes?

Part II: Pedal Interaction

Toggle layered
Toggle 1-at-a-time
Toggle all on, all off

https://miro.com/app/board/uXjVM06Lres=/?share_link_id=91798650876

Post-Part II Interview Questions

- How did you feel about the pedal interaction? Was it difficult to navigate? How would you improve this?

A2. Ranking Boards created in Miro for Ranking task

Part I: Task Conditions (8 Combinations of Contour Style and Highlight Style)

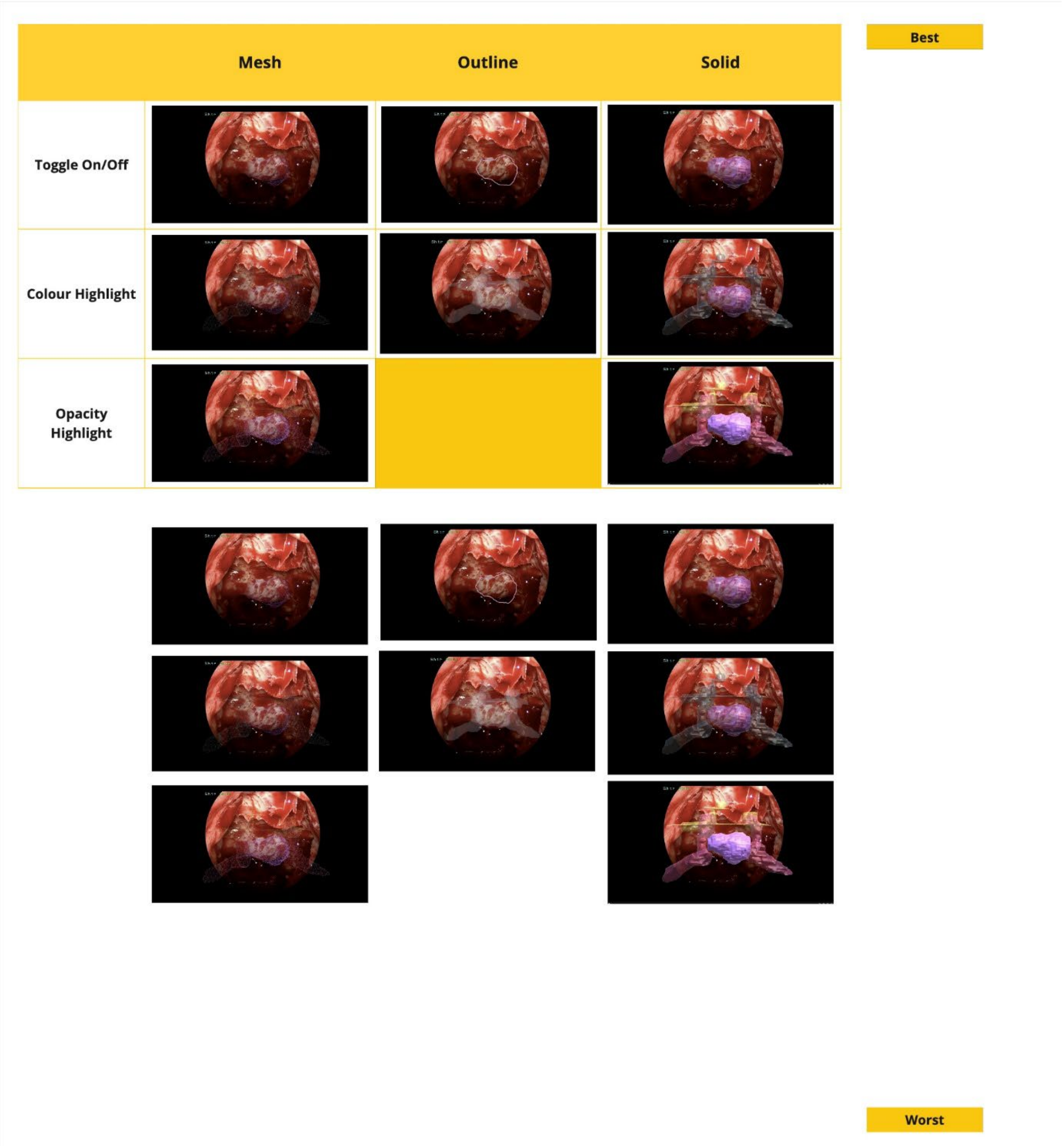

Post-Part I Interview Questions

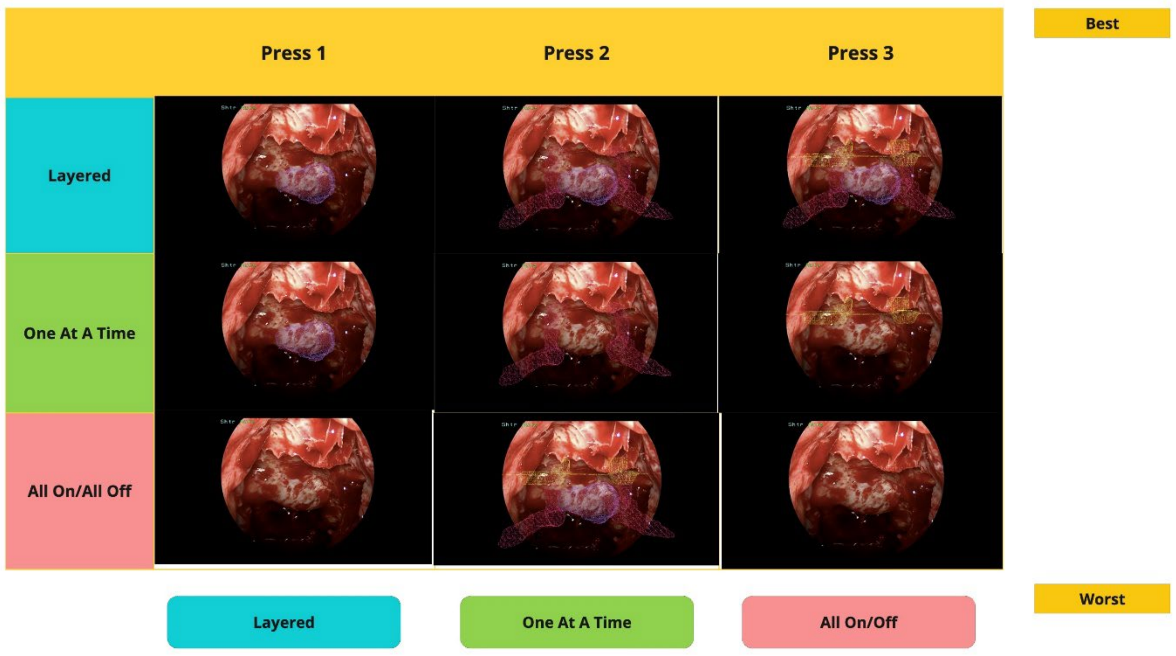

Supplement: Supplementary file 1 — (pdf 381 KB) [file 11548_2024_3225_MOESM1_ESM.pdf]
