# Supplementary material for: Augmented reality for endoscopic transsphenoidal surgery: evaluating design factors with neurosurgeons
Source: Int J Comput Assist Radiol Surg. 2024 Jul 26;20(1):131–6. doi: 10.1007/s11548-024-03225-9 (PMC11759473; doi:10.1007/s11548-024-03225-9)
Supplement: Supplementary file 3 — (pdf 9696 KB) [file 11548_2024_3225_MOESM3_ESM.pdf]

Study 1: Semi-structured Interview Guide

The research I am conducting is an inquiry into the the sellar phase of the endoscopic transsphenoidal approach.

This stage has been selected as it has been observed to warrant corroboration between adjuncts for navigation and confirmation.

In our conversation today, I am keen to better understand details of the techniques and navigation tools you employ at different steps to navigate around anatomical features and landmarks.

My hope is to apply this information to design an augmented reality (AR) tool for the endoscopic transsphenoidal approach.

A brief introduction to augmented reality, the potential for artificial overlay and the potential for integration with neuronavigation.

Intro Q: Before we begin, perhaps you could tell me a little more about yourself, and your specialisation and role in this type of surgery?

I'll briefly introduce each step in the sellar phase just to be sure we're discussing the same step. Please feel free to comment, extend or correct any of this information, because you are certainly the expert.

An inquiry into the 'Confirmation of adequate exposure and identification of pertinent landmarks' Step of the sellar phase

At the beginning of the sellar phase, there's the opportunity to visualise landmarks inside the sphenoid sinus. I have a photo here that we could analyse together.

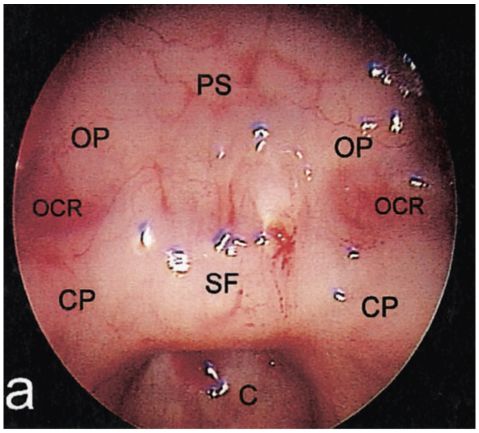

Q1: What information helps you determine the entry point? Perhaps some of them are particularly important?

An inquiry into the 'Sellotomy' step of the sellar phase

From the medical literature I understand that, when the sella is extended it is guided by the tumour behind the sella.

Q2: How do you accurately determine how much you should extend given that the tumour is behind the sella?

Q2.1: If augmented reality was to be applied to the tumour behind the sella, do you think this would aid in accurately determining the extension required?

An inquiry into the 'Confirmation of adequate exposure and identification of sella limits and neurovascular landmarks (e.g. optic nerves and carotid arteries)' step of the sellar phase.

The application of an ultrasound doppler in the sellar phase is helpful for the identification of the carotid arteries, which allows for a safer opening of the dura.

Q3: How do you use the doppler to determine the fine edges of the carotid arteries for a safer sellotomy and durotomy?

Q3.1: If augmented reality was to be used to visualise the carotid arteries, do you think it could help?

An inquiry into the 'Confirmation of adequate exposure and identification of sella limits and neurovascular landmarks (e.g. optic nerves and carotid arteries)' step of the sellar phase

Visualisation of the optic nerves in the sellar phase is also important in preventing optic nerve injury.

Q4: How do you determine the fine edges of the optic nerves for a safer durotomy?

Q4.1: If augmented reality was to be used to visualise the optic nerves, do you think it could be helpful?

An inquiry into the 'Durotomy' Step of the sellar phase

From what I've read, the dura is incised in a midline position. Is that accurate?

Q5: Why is it important to maintain a midline position and how do you determine the midline?

An inquiry into the 'Durotomy' Step of the sellar phase

From my understanding, in smaller tumours, the sellar dura may be covered in one or two venous channels which can bleed during the dural incision. And to avoid this, a small dural incision is initially performed until the venous sinus is reached, secured and sealed with bipolar coagulation forceps or with two surgical clips before the dura can be safely incised.

Q6: Have you encountered such a case in your surgical practice?

Q6.1: How do you go about locating the venous sinus?

An inquiry into the 'Durotomy' Step of the sellar phase

From my understanding, in patients whose bodies produce an excess amount of growth hormone, portions of the carotid artery can become dilated.

Q7: How do you use the tools in the OR to accurately navigate around such cases in order to proceed safely?

An inquiry into the 'Microadenoma & Microadenoma resection' steps of the sellar phase

I've read that, in tumour removal there are instances where there are challenges in identifying the tumour from the pituitary tissue.

Q8: Could you help me understand how you navigate such instances?

An inquiry into the 'Macroadenoma: piecemeal resection' step of the sellar phase

From my understanding, the removal of larger tumours, i.e. macroadenomas, may require a stepwise approach that begins with the removal of the lower fragments, the fragments on the sides, followed by the upper fragments.

Q9: How do you accurately determine the lower, lateral (the fragments on the sides) and upper aspects of the tumour in order to effectively perform this manoeuvre?

An inquiry into the 'Confirmation of adequate resection' step of the sellar phase

Q10: Once the tumour has been removed, how do you compare the video imaging from the endoscope with the neuronavigation tool to confirm total or adequate removal?

Q11: Is there anything you would like to discuss that we may have not touched upon in this interview?

Thank you so much for being a part of this study.

Study 1: Preliminary Evaluation Think-aloud Interview Guide

A few weeks ago we spoke about the information you look for during the sellar phase of the endoscopic transsphenoidal approach. This is a follow-up session that is anchored in that discussion.

We have developed a simple prototype to assist with the overlay of various structures. As we discuss elements of the prototype, we will assume that we are at various steps of the sellar phase so I can better understand what is useful in the surgical workflow. At any stage you are absolutely welcome to reference the use of this overlay together with the neuronavigation or the ultrasound doppler. And when you need to visualise something I'll do it for you because in theatre you would be holding an endoscope as well as other instruments.

I'd be quite keen to know your thought process with each visualisation request, for example it would be great if you let me know: *"I am now in the sphenoid sinus, I need to know where this structure is so that I can ensure that this happens / doesn't happen."* So it's more like you expressing your inner dialogue.

With this prototype we can visualise the carotid arteries, the cavernous sinus, the optic nerves, and the tumour. You can show or hide the overlay of individual structures, or overlay them on top of one another as you see fit.

Demonstrate visualising the different structures.

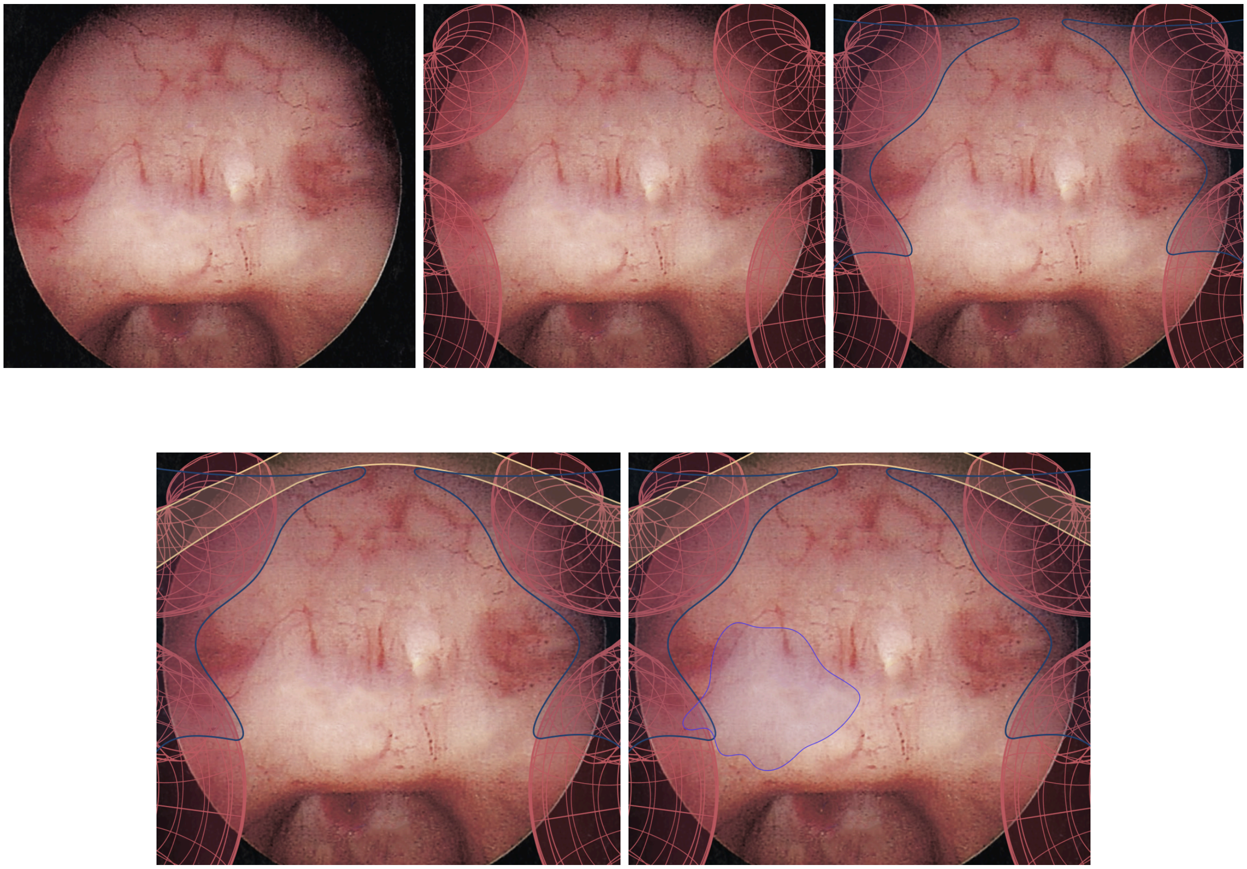

An evaluation of overlay during the 'Confirmation of adequate exposure and identification of pertinent landmarks' Step of the sellar phase

Q1: Assuming we are within the sphenoid sinus looking at the skull base bone. Is there anything you would like to visualise to guide you at this step? And could you talk me through it?

Show/Hide the visualisations as per their request

An evaluation of overlay at the 'Sellotomy', 'Confirmation of adequate exposure and identification of sella limits and neurovascular landmarks (e.g. optic nerves and carotid arteries)' & 'Durotomy' steps of the sellar phase

Q2: We have now identified the sella and would like to remove the bony tissue. Is there anything you would like to visualise to guide you at this step? And could you talk me through it?

Show/Hide the visualisations as per their request

Q3: We have now removed the bony sella tissue. Could you please walk me through how you would proceed after the bony tissue is removed and perhaps talk me through your thought process.

Show/Hide the visualisations as per their request

Q4: I have a hypothetical situation with two different sizes of tumours (small and large), and I was curious on how your exposure strategy would vary between the two if you had information on their size and location with the overlay like so...

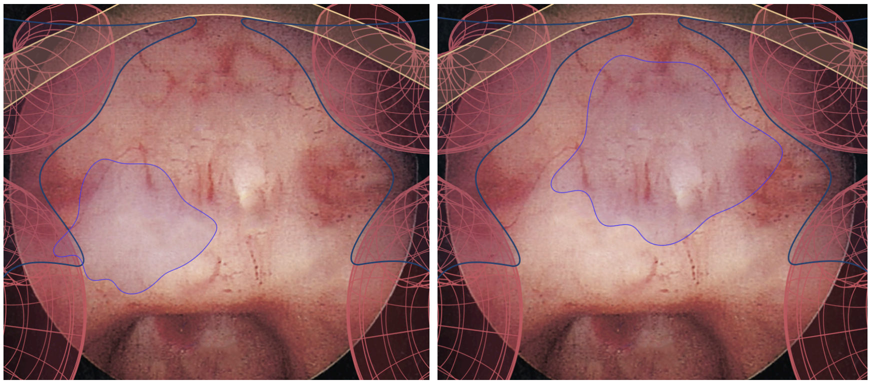

Note on Q4:

There was tension in the data from the semi-structured interview on sella exposure strategies and the need to visualise the tumour with AR. One strategy involved minimal exposure guided by the location and size of the tumour while the other was more extensive and did not necessarily look to the location or size of the tumour as a guide for exposure. Presenting scenarios in the evaluation of the mock-up - where the location of the tumour varied - was, therefore, important to confirm and understand this finding.

A tumour that slightly invaded the cavernous sinus on one side was chosen as a scenario [https://www.ncbi.nlm.nih.gov/pmc/articles/PMC5463411/]. For this, the Knosp grading system for pituitary tumours was referenced and a grade 1 chosen primarily to justify skewing the tumour to one side. For a different scenario, one that was more central and in the suprasellar region was chosen [https://link.springer.com/article/10.1007/s11102-020-01043-1]. Based on the medical literature, these are potential manifestations for pituitary adenomas. These manifestations, therefore, provided justification to position AR overlay of the tumour in these positions to further investigate surgeons' exposure strategies.

Different sizes were chosen as tumours also tend to manifest as microadenomas or macroadenomas. Presenting a variance in size for different scenarios was an investigation strategy to elicit a richer dataset that could be useful in developing a well-rounded array of design requirements.

Feedback on the prototype

Q5: Based on this simulation, how would you say the tool can be improved?

Study 1: A Selection of Encoded Quotes from Participant Surgeons

Participant Number	Context	Quote
P2	**Semi-structured Interview**<br><br>Using bony contours as a primary strategy for discerning the dense network of underlying structures	*"The first thing is identifying the sella, which tends to be a bulge. You look at the contours and the gradient of the bone. Sometimes you can see the optic-carotid recesses, which are little indentations lateral to the sella. From the optic-carotid recesses one can work out the optic protuberance and the paraclival carotid arteries. The planum and the tuberculum above the sella, tend to be an indent-in and a contour-out. The clivus is normally quite obvious, it's a recess and you can see the shadow. This all varies, some cases are difficult. [...] You want to avoid opening the carotids and the optic nerves. AR could highlight these structures and provide the utility of safety."*
P3	**Semi-structured Interview**<br><br>Challenge in determining the limits set by the surrounding critical structures during sella exposure	*"What's harder to be sure of is where the limits of the sella are laterally, particularly when the bone is thick. [...] The most useful thing to overlay would be the optics and the carotids because those are structures I want to avoid. I'm happy to take bone up near to them, so AR would help me decide how far to push it."*

P1	**Semi-structured Interview**<br><br>Description of exposing the sella in a minimal fashion	*"One uses neuronavigation to work out how much to open, you'll be limited by the anatomy around, so you don't want to go too far lateral and damage the carotids, for example. On the flip side, you probably want to do as minimal opening of the sella as possible to get you the optimal access, which I think could partly be judged by whether you feel like you could access the tumour."*
P4	**Semi-structured Interview**<br><br>Description of exposing the sella in a minimal fashion	*"It would be nice to know where the tumour generally is with AR, I would probably check that at each stage. If you have something available, why not plan and why not try and make the opening smaller and smaller?"*
P3	**Semi-structured Interview**<br><br>Description of exposing the sella more extensively giving priority to instrument manoeuvrability	*"I'd be happy to remove as much bone as possible, even if the tumour is not very big. You want to have reasonable exposure to get instruments into the sella. Overlaying the carotids and the optics would help me decide how far to push exposure."*
P4	**Mock-up Evaluation**<br><br>Description of how a surgeon would utilise AR in the 'Sellar Phase'	*"With the tumour overlay I've got an idea of how big of a sellotomy I'll need to make. With the overlay of the neurovascular structures I know that this area is dangerous (points to the lateral margins of the carotids and cavernous sinus) and this area is*

		dangerous (points to the optic nerves superiorly). I would confirm that with the neuronavigation, then probably open up the sella here (points to a central region). I would confirm with neuronavigation, toggle the tumour overlay off, and do the sellotomy."
P3	**Mock-up Evaluation**<br><br>A surgeon's strategy on where to expose the sella for a left-sided tumour highlighted using AR during the mock-up evaluation session	*"I can see quite clearly that the tumour is more on one side than the other – so on the left, probably the exposure on the right doesn't need to be right up to the carotids and doesn't need to be, therefore, as risky. But I need a wider exposure on the left-hand side for this left-sided tumour."*
