# Supplementary figures and images for: Augmented reality for endoscopic transsphenoidal surgery: evaluating design factors with neurosurgeons
Source: Int J Comput Assist Radiol Surg. 2024 Jul 26;20(1):131–6. doi: 10.1007/s11548-024-03225-9 (PMC11759473; doi:10.1007/s11548-024-03225-9)

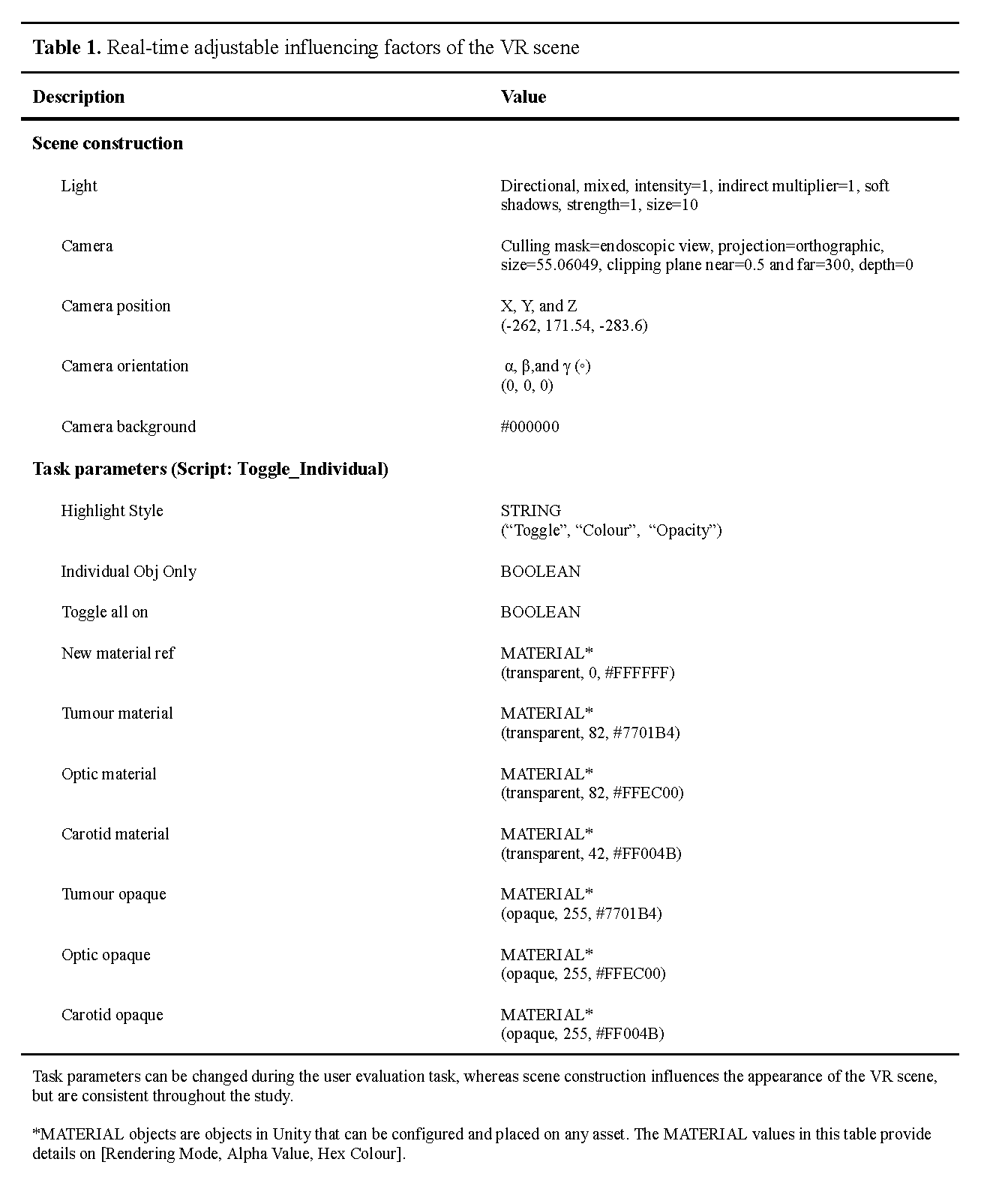

Supplement: Supplementary file 4 — (png 51 KB) [file 11548_2024_3225_MOESM4_ESM.png]
